# Poor Knowledge and Practices Related to Iodine Nutrition during Pregnancy and Lactation in Australian Women: Pre- and Post-Iodine Fortification 

**DOI:** 10.3390/nu4091317

**Published:** 2012-09-20

**Authors:** Karen Charlton, Heather Yeatman, Catherine Lucas, Samantha Axford, Luke Gemming, Fiona Houweling, Alison Goodfellow, Gary Ma

**Affiliations:** 1 School of Health Sciences, University of Wollongong, Wollongong, NSW 2500, Australia; Email: hyeatman@uow.edu.au (H.Y.), cjl623@uowmail.edu.au (C.L.); samaxford@hotmail.com (S.A.); l.gemming@ctru.auckland.ac.nz (L.G.); fionahouweling@yahoo.com.au (F.H.); 2 Illawarra Shoalhaven Local Health District, NSW Health, Wollongong, NSW 2500, Australia; Email: goodfellow@sesiahs.health.nsw.gov.au; 3 Institute of Clinical Pathology and Medical Research, Westmead Hospital, Sydney, NSW 2006, Australia; Email: garyma@gmail.com

**Keywords:** iodine, supplementation, fortification, pregnancy, lactation

## Abstract

A before-after review was undertaken to assess whether knowledge and practices related to iodine nutrition, supplementation and fortification has improved in Australian women since the introduction of mandatory iodine fortification in 2009. Surveys of pregnant (*n* = 139) and non-pregnant (*n* = 75) women in 2007–2008 are compared with surveys of pregnant (*n* = 147) and lactating women (*n* = 60) one to two years post-fortification in a regional area of New South Wales, Australia. A self-administered questionnaire was completed and dietary intake of iodine was assessed using a validated food frequency questionnaire. A generally poor knowledge about the role and sources of iodine in the diet remained after fortification. Post-fortification, iodine-containing supplements were being taken by 60% (up from 20% pre-fortification) and 45% of pregnant and lactating women, respectively. Dairy foods were the highest contributors to dietary iodine intake (57%–62%). A low intake of fish and seafood resulted in this food group contributing only 3%–8% of total intake. A low level of public awareness regarding the role of iodine in health supports the need for public health strategies in addition to fortification, such as an accompanying consumer education campaign, increased uptake of supplementation, and on-going monitoring.

## 1. Introduction

Iodine deficiency is one of the most common nutrient deficiencies in the world, with almost one billion people affected. Inadequate iodine intake during pregnancy is of particular concern as, depending on the severity of deficiency, it can result in miscarriages, stillbirths, cretinism, irreversible mental retardation, impaired psychomotor development, and behavioural problems [1,2,3]. Iodine requirements are increased from an RDI value of 150 µg/day in adolescents and adulthood, to 220 and 270 µg/day in pregnancy and lactation, respectively [4]. Increased requirements are due to (1) an increase in the production of thyroxine (T4) by the mother to maintain her euthyroid state and transfer of thyroid hormone to the foetus, (2) transfer of iodine from mother to foetus and (3) an increased renal iodine clearance by the mother [1].

Meta-analyses indicate that moderate to severe iodine deficiency without supplementation may result in a population-level loss of intelligence in children of around 10–13.5 IQ points [5,6]. Implications of mild iodine deficiency are less well defined, however developmental gains have been demonstrated in New Zealand children aged 10–13 years following correction of iodine deficiency through supplementation with 150 µg iodine per day [7].

Australia has been classified (prior to mandatory iodine fortification in 2009) as mildly iodine deficient by the World Health Organisation (WHO) [8]. Numerous studies have shown that children and pregnant and breastfeeding women are at high risk [9,10,11,12,13,14]. In Australia, as elsewhere, causes of iodine deficiency are related to low levels of iodine in foods grown in soils that are deplete of iodine, coupled with low intakes of iodine-rich fish and seafood [15] and minimal use of iodised salt in commercially produced foods [16]. Iodised table salt is available through voluntary fortification by salt producers but its uptake is low [17]. Other suggested reasons for the increasing prevalence of inadequate iodine intakes are the widespread replacement of iodophors with other cleansing agents in the dairy industry [18] and a general lack of awareness within the population about the importance of iodine in the diet [19].

Mandatory fortification of salt used in the bread-making process was introduced in Australia in 2009 [20]. In 2010 the National Health and Medical Research Council released a public statement that recommends iodine supplementation (150 µg/day) for pregnant and breastfeeding women, in recognition that mandatory fortification of bread would not meet the increased needs of pregnant and breastfeeding women [21]. This paper explores whether the knowledge and practices related to iodine nutrition have changed since these two public health strategies were introduced, and investigates dietary intakes of iodine, in an attempt to better understand the need for nutritional and supplementation advice targeting these groups.

## 2. Experimental Section

Cross sectional studies undertaken in the Illawarra region of New South Wales, Australia, before introduction of mandatory fortification in pregnant (*n* = 139) [13] and non-pregnant (*n* = 78) [19] women (2007–2008) are compared with studies conducted 1–2 years post fortification (2011–2012) in pregnant (*n* = 147) [22] and lactating women (*n* = 60) [23,24]. Pregnant women were sampled from a single public antenatal clinic in Wollongong, about 80 kilometres south of Sydney. Pregnant women across all three trimesters were invited to participate in the study by clinic staff as they attended for routine antenatal appointments. Non-pregnant women were recruited from workplaces through advertisements and email correspondence. Exclusion criteria included major illnesses, use of antihypertensive medication and/or diuretics, pregnancy or lactation, and formal training in nutrition. Lactating mothers within their first six months of breastfeeding were recruited from seven early childhood centres that operated within the Sydney-based health services. In all surveys, non-English speaking women were excluded. 

Consenting women provided a spot urine sample to determine median urinary iodine concentration (MUIC) [22], completed a self administered questionnaire on supplement use, iodine knowledge and practices, and in the post-fortification surveys, a self-administered validated iodine specific food frequency questionnaire [25] was completed (note that the validation was with an elderly population, not young women). The FFQ asked about intake over the past month of the food categories dairy, eggs, cereal products including bread, fish and seafood, meat, vegetables, fruit and mixed dishes. It also contained questions about tap water consumption and use of iodised salt. Food intakes were entered into a computerised dietary assessment package (FoodWorks version 6.2, Xyris Software, Highgate Hill QLD) to assess iodine intakes, based on the AusNut 2007 nutrient composition database [26]. 

The questionnaire was adapted from a New Zealand [27] and South African study [28] and modified for use in pregnancy or lactation, where necessary. Questions related to knowledge of iodine nutrition included health implications associated with inadequate iodine in the diet and sources of dietary information. Questions related to practices included changes made to the diet with the intention to increase iodine intake, reported nutritional supplement use (brandname and doseage), and the use of iodised salt. Participants were asked about their current level of awareness of iodine as a public health problem and whether they felt confident they had received enough information to make informed choices on various nutrition concerns (during pregnancy/lactation). Information on the number of previous pregnancies and/or miscarriages was also obtained. Post 2009, questions were added relating to awareness of the mandatory iodine fortification programme.

Approval for all of the surveys was granted by the University of Wollongong Human Research and Ethics committee and the South East Sydney Illawarra Area Health Services.

## 3. Results

Characteristics of women included in the four surveys are shown in Table 1. MUIC results are reported elsewhere [22], only results relating to knowledge and dietary practices, including factors associated with supplement use are provided here. 

**Table 1:** Sample characteristics including age, pregnancy trimester, previous miscarriage and education level.

Study (Year)	Survey (Year)			
	Pre-Fortification		Post-Fortification	
	Non-Pregnant [19]	Pregnant [13]	Lactating [23,24]	Pregnant
	(2007–2008) *N* = 76	(2008) *N* = 139	(2010) *N* = 60	(2011) *N* = 147
**Age **				
Mean (SD)	38.3 (10.6)	28.4 (5.7)	32 (3.9)	28 (5.0)
Range	19–56	16–45	23–39	16–40
**Highest Level of Education Attained**				
Some High School	-	17%	-	23%
Completed High School (Year 12)	13%	30%	13%	19%
TAFE (Technical and further education or apprenticeship)	21%	31.00%	17%	32%
University Degree (Undergraduate Level)	32%	13%	38%	21%
University Degree (Postgraduate Level)	35%	9%	32%	6%
**Trimester**				
1 (0–12 weeks)	-	2%	-	2%
2 (13–24 weeks)	-	13%	-	37%
3 (>25 weeks)	-	85%	-	61%
**First Pregnancy/Birth**	-	41%	88%	46%
**Intention to Breastfeed**	-	84%	All breastfeeding	88%
**Previous Miscarriage**	-	29%	-	25%

### 3.1. Supplement Use

Pre-fortification, 59% of women indicated supplement use during their pregnancy, but only 20% were taking supplements containing iodine. Nutritional supplement use was higher among women in their first pregnancy (72% *vs.* 49%; *p *< 0.05) and women who had a tertiary education (74% *vs.* 54%; *p* = 0.049). Most of the supplement users had been taking them for over 3 months (3–6 months, 36.5%; >6 months, 41%). Post-fortification, nutritional supplement use was 71% and 58% in pregnant and lactating women, respectively. Iodine-containing supplements were being taken by 60% and 45%, respectively. Most of the breastfeeding women had been taking the supplements for longer than six months (6–9 months, 13%; 9–12 months, 22%; >12 months, 17%). 

### 3.2. Knowledge

In all surveys, knowledge about iodine was generally poor across all areas assessed. Post fortification, the majority of pregnant women did not know if their diet provided adequate iodine for their own bodies’ needs (64%) nor for their unborn child’s needs (68%). Only 5% of women correctly identified bread as legally required to have iodine added.

**Figure 1:**
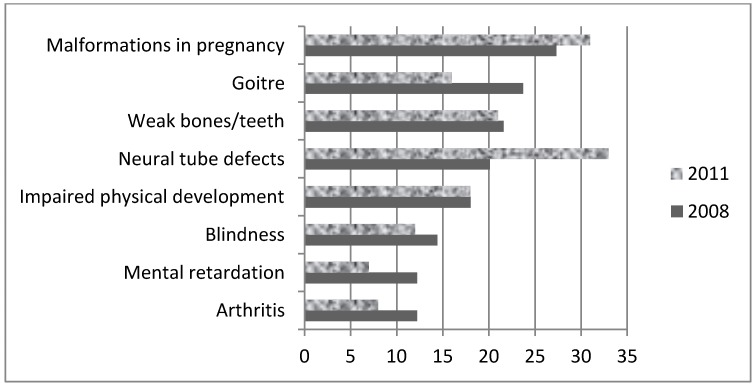
Knowledge of adverse consequences associated with iodine deficiency: Pre- (2008; *N* = 139) and Post- (2011; *N* = 147) fortification: % pregnant subjects who responded affirmatively when asked if the condition was related to iodine deficiency.

**Figure 2:**
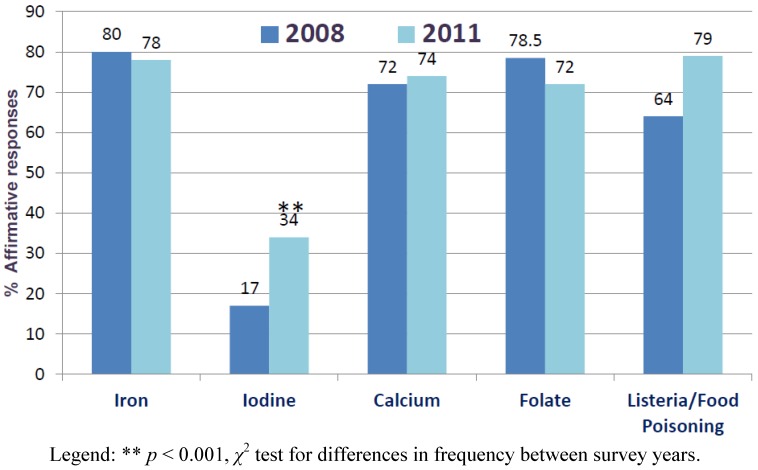
Pregnant womens’ responses regarding whether they had received sufficient dietary information on nutrition while pregnant to make informed decisions: Pre- (2008) and Post-fortification (2011).

Knowledge of iodine rich food sources was lacking both before and after mandatory fortification; 50%–58% of women correctly identified seafood as a good source while between a quarter and a third correctly identified milk, eggs or bread (Table 2). Participants were also limited in their ability to identify outcomes related to iodine deficiency and at risk groups; between 27% and 31% of pregnant and lactating women correctly identified mental retardation as a potential outcome (Figure 1). Both pre- and post-fortification, most women did not know if iodine was a public health problem in Australia (76%–90% *vs*. 80%–85%, respectively). Although some improvement following fortification was evident, most women perceived that they had not received enough information regarding iodine to make informed decisions during their pregnancy (83% (pre-) and 66% (post-fortification)). Women were much more confident about their knowledge of other nutrition related topics in pregnancy, including healthy eating, listeria and food poisoning, folate, calcium and iron (See Figure 2). The most commonly cited source for nutrition advice, including iodine, across all surveys was verbal information from healthcare professionals, followed by written information from healthcare professionals and the internet (Table 3).

**Table 2:** Knowledge about iodine in pregnant women pre- and post-fortification (response to question: “Which foods are good sources of iodine in the Australian diet?”) ^a^.

Food Source	Good Source ^b^		Do not know	
	Pre- ( *n* = 139)	Post- ( *n* = 145)	Pre- ( *n* = 139)	Post- ( *n* = 145)
Meat	49%	50%	48%	48%
**Milk** *	**26%**	**26% **	64%	63%
**Bread** * **^,c^**	18%	**27%**	72%	61%
**Fish and Seafood** *	**58%**	**50% **	39%	43%
Fruit	37%	27%	58%	65%
Vegetables	59%	55%	39%	42%
**Eggs** *	**23% **	**32% **	67%	63%
**Salt** * **^,d^**	**52%**	**51% **	36%	40%

Bold text: correct responses; * Good sources of iodine; ^a^ Identified affirmatively; ^b^ Considered good source if the food contributed >5% of total iodine intake in the Food Standards Australia New Zealand (FSANZ) 22nd Australian Total Diet Survey (2008) [29]; ^c^ Fortified only after 2009; ^d^ Only good source if iodised.

**Table 3:** Numbers of pregnant women (*n*) reporting particular sources of advice for different nutrition related topics (pregnant women 2011; *n* = 145) (adapted from [13]).

Source of Advice	Iron	Iodine	Calcium	Healthy Eating	Folate	Listeria and Food Poisoning
**Healthcare Professional Advice (Verbal)**	72	53	81	101	75	99
**Healthcare Professional Advice (Written)**	48	18	31	52	51	43
**Newspaper**	3	1	2	3	3	4
**Magazine**	18	7	19	20	12	14
**Television**	7	1	7	7	7	4
**Radio **	0	0	1	0	1	0
**Internet**	23	11	16	27	19	32
**Did not receive any information**	24	51	26	8	20	16

### 3.3. Practices

Practices related to iodine that were assessed included dietary changes related to iodine and use of iodised salt. Between 11% (pre-fortification) and 22% (post-fortification) of women reported they had made changes to their diet to increase iodine intake since becoming pregnant. Most commonly reported dietary changes included increasing seafood intake and switching to iodised salt. Some women reported an increased consumption of foods, such as meat and vegetables that would make little difference to iodine intake. 

Iodised salt use, in cooking and/or at the table, was similar pre- (pregnant women, 40%) and post-fortification (pregnant, 49%; lactating, 45%) but non-pregnant women reported a higher use of iodised salt in the 2007–2008 survey (60%). 

Mean dietary iodine intake calculated from the FFQ was 176 (SD = 92) µg/day (range 21–463 µg/day) for pregnant women (*n* = 130) and 146 (SD = 58) μg/day (range 43–342 µg/day) for lactating women, which increased significantly when iodine from mandatory fortification of bread was calculated (Table 4). For both pregnant and lactating women, milk and dairy foods were the highest contributors to iodine intake (57%–62%), followed by cereal products including bread (19%–21%), tap water (7%), seafood (3%–8%) and eggs (3%–4%). Other foods such as meat, fruit, vegetables and mixed sources, including take away foods, contributed a total of 2%–5% iodine intake. In pregnant women, iodised salt contributed 8% of dietary iodine (not calculated in lactating group).

**Table 4:** Dietary intake of iodine post-fortification in pregnant and lactating Australian women.

Total iodine intake (Mean ± SD)	Lactating (2010) [24] *N* = 60	Pregnant (2011) *N* = 130
EAR	190 µg/day	160 µg/day
With bread	182 ± 63 μg/day	211 ± 98 µg/day
Without bread	146 ± 58 μg/day **	176 ± 92 µg/day *
% <EAR (with bread)	60%	35%

** *p* < 0.001; * *p* < 0.05; for differences between iodine intake estimations with and without bread.

## 4. Discussion

Despite recommendations that all pregnant and lactating women in Australia should take supplements containing 150 µg iodine per day [21], 40% of pregnant women and 55% of lactating women in the current study were not receiving iodine supplementation. However, the use of supplements containing iodine had increased from 20% to 60% in pregnant women, pre and post fortification, an important improvement. Prior to 2009, the major commercial brand of pregnancy supplements in Australia did not contain iodine [30] which explained our earlier finding of only 20% uptake in pregnant women [13]. The reformulation of pregnancy supplements to include appropriate levels of iodine reflects a positive move by industry and should be further encouraged until all supplements for pregnant women contain appropriate levels of nutrients, including iodine.

We have previously reported data from the current study sample that 1–2 years following introduction of the mandatory iodine fortification programme of salt used in bread-making in Australia, median urinary iodine concentrations of both pregnant and lactating women indicated sufficiency only in those who were taking iodine-containing supplements [23,24].

Since the early 1990s, there has been little public debate in Australia around issues of nutrient fortification of foodstuffs in the food supply [31] and this may contribute to lack of awareness of such public health activities. To the authors’ knowledge, no public or professional education initiatives directed to the importance of iodine during pregnancy and lactation have been conducted following the introduction of mandatory iodine fortification or the release of the NHMRC position statement on the need for iodine supplementation for pregnant and lactating women, at least not in the area of New South Wales where this work was conducted. Our data were the first to report that Australian women have a low level of knowledge regarding the detrimental outcomes of iodine deficiency during pregnancy [13,19], and this has not changed after fortification. Indeed, few women are aware that bread is required by law to be fortified with iodine. 

Many women incorrectly identified neural tube defects and weak bones and teeth to be related to iodine deficiency, which suggests general confusion between the function of iodine with other micronutrients. The simultaneous implementation of both folic acid and iodine fortification of bread in Australia [20] may have contributed to this consumer misunderstanding. 

Women’s access to information about iodine in pregnancy and lactation remains inadequate post-fortification. Although the percentage of women who believed they had received adequate information about iodine had doubled since the 2008 survey [13], it was still far lower than information received regarding other health and nutrition priorities in pregnancy. In response to the findings of our 2008 study in pregnant women, there has been a change in clinical practice in the public antenatal facility that was sampled, whereby all pregnant women should now receive an information sheet about iodine at their first clinic visit. Despite this, most women remained uninformed about the need to ensure an adequate iodine intake during pregnancy and lactation and only approximately half (49%) of the pregnant women reported receiving verbal or written information from a healthcare professional. Many women receive early antenatal care from their general practitioners and may not attend antenatal services until later in their pregnancy. In either situation, limited importance may be given to verbal communication from the health care practitioner, as reported by the women, compared with distribution of printed materials alone.

There was also confusion about food sources of iodine, with many women identifying vegetables and meat as good sources. Although the majority of women identified fish and seafood as being rich sources of iodine, few were consuming seafood regularly, as evidenced by its low contribution to total iodine intake (3%–8%). Concerns regarding mercury contamination in seafood and the risk of listeriosis and other food poisoning agents may be deterring pregnant women from consuming larger amounts of seafood, and leading to the omission of this important nutrient-rich food group [32,33]. Further qualitative research regarding factors that influence fish intake in pregnancy is warranted, to better understand the way in which pregnant women assimilate the various nutrition-related messages that are provided simultaneously. Indeed, participants were better informed regarding iron, folate, calcium, *Listeria* and food poisoning, and were more confident that they could make informed decisions regarding these issues, compared to food choices about iodine. Information on iodine is provided to pregnant women attending public antenatal clinics in the state in which the present surveys were completed (*i.e.*, New South Wales) [34] however the information is provided in a general booklet that also includes information on folic acid, food safety and mercury in fish. Materials are also available on various government and non-governmental websites [35,36,37,38].

Our studies confirm that dairy foods remain the major contributor to total iodine intake, due to their high frequency of intake, even despite changes in dairy industry sanitation practices in Australia, whereby iodophors have been replaced with other sanitizing agents [18]. This study shows intakes increased by approximately 35 μg/day after the iodisation of bread, which is in line with the estimated increase of +46 μg/day in adults, that was modelled by Food Standards Australia New Zealand (FSANZ) in their assessment report [17].

This paper reports on findings relating to studies of women in one region of New South Wales therefore generalizability to other states and geographical areas, such as inland locations, cannot be made. Self report bias of participants completing the questionnaire and food frequency questionnaire cannot be ruled out. However, the use of standardized instruments across surveys, together with a lack of change in knowledge and awareness over time, supports our conclusion that women are no better informed about iodine following introduction of one of only three mandatory nutrient fortification programmes in the country introduced for public health outcomes, the others being thiamin (1991) and folic acid (2009) also added to bread. 

## 5. Conclusions

The combination of inadequate knowledge regarding iodine and the limited use of iodine supplements in pregnancy and lactation highlights a potential public health issue of concern in Australia. Mandatory fortification may be overcoming this knowledge and behaviour deficit to some degree, however dietary intake alone is insufficient to meet the increased iodine needs of women during pregnancy and lactation. Strategies within antenatal and postnatal services are required to improve iodine supplementation practices in women. 
